# Re-Audit of the Physical Health Monitoring of Patients Receiving Antipsychotic Treatment in Ty Llywelyn Medium Secure Unit

**DOI:** 10.1192/bjo.2025.10541

**Published:** 2025-06-20

**Authors:** Justina Akinlua

**Affiliations:** BCUHB, Llanfairfechan, United Kingdom

## Abstract

**Aims:** The aim of conducting this audit is to know whether the physical health monitoring of our clients are done appropriately in accordance with the BCUHB guidelines for physical health monitoring for adults prescribed antipsychotic therapy and also to reduce the risk of adverse side effects of antipsychotics on the physical health of our patients.

**Methods:** The sample in this audit consists of 18 patients (n=18) in Ty Llywelyn. To collect the data, we utilised the patients’ paper files, Paragon (computerised) clinical entries, drug charts and observation charts. The participants also had their waist circumference measured using tape measurement. Data collection was undertaken in June and July 2024 using the audit proforma. Completed GASS questionnaires were filed in the patients’ notes and the prescriptions initiated because of the questionnaires were accompanied by a Paragon entry to explain the rationale.

**Results:** In this re audit, it was noticed that most of the patients had their waist circumference measured. Whereas in the previous audit, waist circumference was not done on the patients. Out of the 18 people who participated in the audit only 17 people had their waist circumference measured. Out of the 17 people, 13 patients were shown to have obesity according to their waist circumference and 3 patients fall within the overweight range of waist circumference. Only one patient’s waist circumference falls within the range of normal. In addition, in this audit, we identified that 12 out of the 18 patients who participated in this audit have normal HBA1C meaning that they are not diabetic, while 6 out of the 18 patients are diabetic. Of the 18 participants in this audit, a total of 2 patients had normal weight. A total of 11 participants are obese. A total of 1 patient is overweight. A total of 4 patients are severely obese.

**Conclusion:** The results highlight the importance of regular monitoring and the need for targeted interventions to manage obesity, reduce and eliminate diabetes and also to reduce the risk of adverse side effects of antipsychotics on the physical health of our patients. Overall, the physical health of the patients in Ty Llywelyn medium secure unit is taken seriously, hence the higher percentage of people who have their physical health checked in this study.

